# Nursing students’ experiences of workplace violence based on the perspective of gender differences: a phenomenological study

**DOI:** 10.1186/s12912-023-01551-y

**Published:** 2023-10-18

**Authors:** Jun Cao, Hongbo Sun, Ying Zhou, Anqi Yang, Xiaoshu Zhuang, Jiaxian Liu

**Affiliations:** 1 School of Nursing, Guangzhou Medical University, Guangzhou, China; 2 School of Nursing, Guangzhou Medical University, 195 Dongfeng West Road, Yuexiu District, Guangzhou, P.R. China

**Keywords:** Nursing students, Workplace violence, Gender differences, Phenomenological research

## Abstract

**Background:**

Workplace violence is a worldwide concern, and particularly affects nursing students. It has a seriously negative impact on nursing students’ clinical learning experience and their physical and mental health. This study explored whether there are differences in psychological responses and coping styles among different gender nursing students after exposure to workplace violence, and investigated the causes for these differences.

**Methods:**

We enrolled 22 nursing undergraduates from Guangzhou Medical University and Zunyi Medical University, China. Phenomenological qualitative research and online semi-structured interviews were conducted. The data were analyzed by the Colaizzi seven-step content analysis method.

**Results:**

Two categories were collated: psychological experience and coping styles. Three themes of the former were extracted: negative emotional experience, low level of professional identity, and negative effect on self-efficacy. Two themes of the latter: responses to violence and adjustment after violence. In addition, fourteen subthemes were extracted.

**Conclusions:**

Different gender nursing students have different psychological experience and coping styles in the face of workplace violence. The causes of the differences are likely related to sociocultural factors and psychological gender status.

## Introduction

Workplace violence refers to insult, threat, or attack by staff in a work environment, and it brings explicit or implicit challenges to personal safety, happiness, and health [1]. Violence can be classified as horizontal and vertical according to the source [1]. Horizontal violence refers to hostile behavior that occurs toward members of the same group. Horizontal violence among nursing students refers to bullying behaviors that involve physical, verbal, and emotional assault among them [2]. Vertical violence refers to violence among colleagues in different positions hierarchically or violence committed by superiors against subordinates. Vertical violence experienced by nursing students refers to the hostile behavior of instructors, other clinical staff members, and patients or their families [3]. In addition, violence is classified as physical or psychological [1]. Physical violence is a physical injury caused by physical attacks, such as beating and pushing. Psychological violence refers to intentional verbal abuse and sexual harassment by words that lead to damage to spirit and social development [4].

Nursing students frequently experience workplace violence, a phenomenon that has become a concern worldwide [5]. Incidence of workplace violence among nursing students ranges from 42.8 to 98.3%, and the forms involve verbal or physical assaults, racial discrimination, and sexual harassment [3, 6–14]. Nursing students experience negative emotions, loss of confidence, and decreased self-esteem after violence. These reactions can lead to a poor sense of professional identity [7–9]. Currently, there are several qualitative studies on nursing students’ experiences of violence in clinical settings, but most mainly focus on females, and less focused on males [5, 7, 11, 15–17]. The theory of gender differences points out that men and women have different specific behaviors, basic attitudes, and feelings in the same environment [18, 19]. However, little is known whether nursing students of different genders have different feelings, attitudes, and coping styles after workplace violence in clinical settings.

Therefore, we adopted the phenomenological qualitative research to explore the inner experience and coping styles of different gender nursing students after workplace violence, exploring the intrinsic meaning of their behavior. The causes and internal mechanisms were also discussed based on hermeneutic philosophy. That all is the originality of the current study. We expect that the findings will provide a reference basis for nursing managers and educators to develop targeted violence countermeasures.

## Theory

**Fig. 1:**
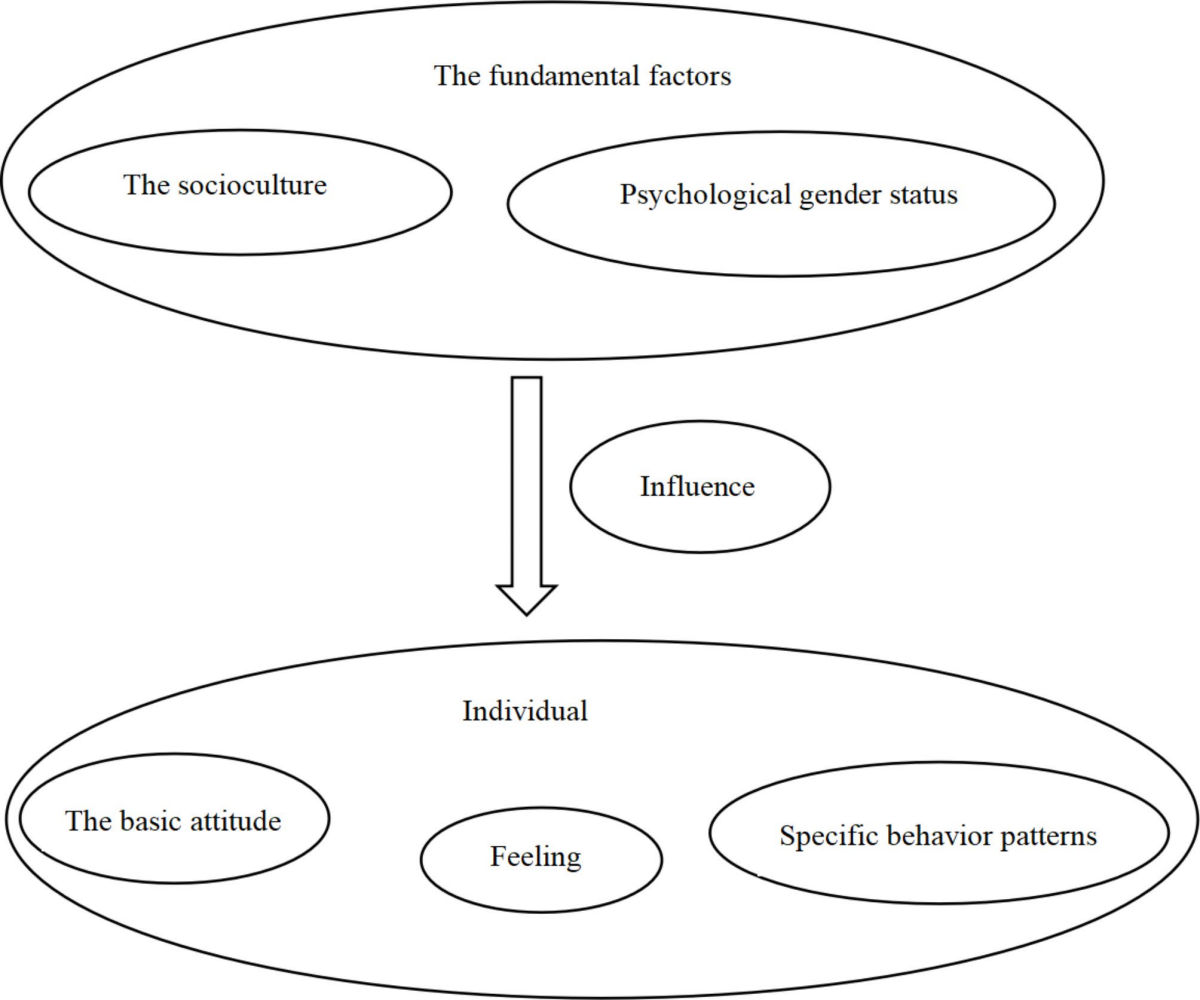
Theory gender differences-----Theoretical framework

**Fig. 2:**
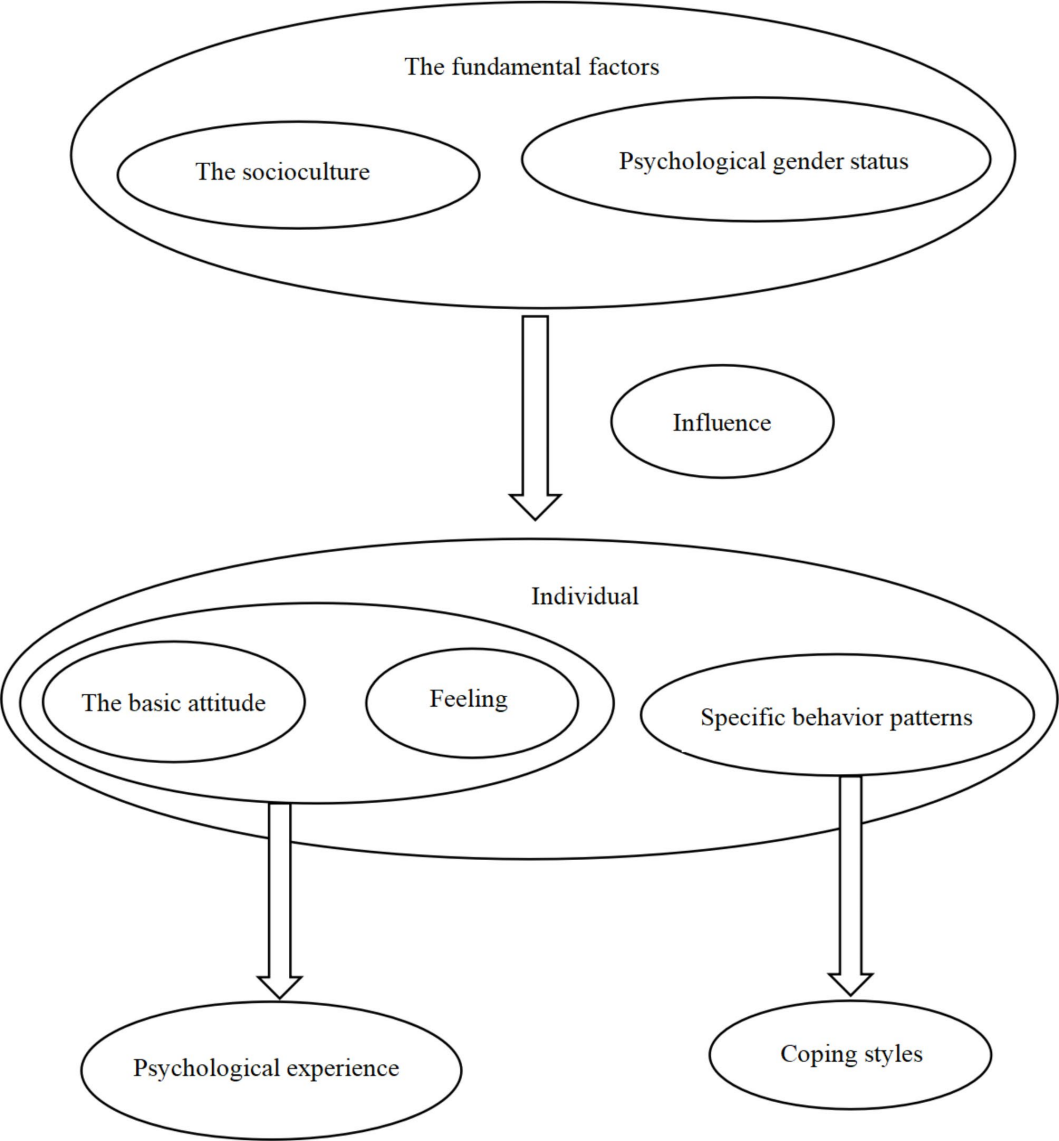
Theory of gender differences-----The research framework

This study was based on the theory of gender differences [18–22]. It originated from the personality gender difference theory in personality psychology. Gender difference theory began in the late 19th century. It examines male and female personalities by correlating gender with behavior. Gender distinguishes male and female individuals based on sociocultural and psychological perspectives. Different cultures have diverse norms and standards for gender. Psychological gender is a specific behavior pattern that individuals can perceive, which is suitable for social cultures, and identifies persons with the mental state of being male or female. In this way, individuals can form a behavioral system, basic attitudes, and feelings that are suitable for social cultures and psychological gender, that is, gender roles. Different gender roles have different specific behaviors, basic attitudes, and feelings that form in a specific sociocultural background and that change with the change of sociocultural factors and psychological gender status [23–25]. The theory of gender differences is used widely in the fields of adolescent violence, mental health, and violence criminal psychology [18, 19]. Figure 1 shows the theoretical framework of gender differences. Figure 2 shows the research framework of this study.

## Methods

### Design

Using the hermeneutic phenomenological method, we observed the individual’s current experiences and fairly described their experiences, and investigated the underlying causes as much as possible [26]. Then a semi-structured, one-to-one, in-depth interview was carried out among the participants. The interview outline was formulated concerning relevant literature [15, 17] and the results of the initial part of this study about workplace violence among nursing students. It mainly found that nursing students’ experiences of violence are mainly related to factors such as personality, gender, internship time, and other factors. The main interview outlines were the following questions:

Do you know about workplace violence? Could you please talk about your understanding?

What aspects of violence have you experienced during your internship? Could you talk about your experiences?

What were your feelings when the violence occurred? Could you further describe a little bit?

How did you handle the occurrence? Why?

How did the violence affect you? How long did the effect of the violence last?

The researchers of this study included one Associate Professor (LJX), one Professor (ZY), two postgraduate nursing students (CJ and SHB), and two undergraduate students (YAQ and ZXS). All of them are females. CJ was the main interviewer and she conducted interviews and primary data analysis. She had 25 months of clinical internship experience and once experienced slight verbal violence from patients four years ago, which had a little negative effect on her. She and the participants work in different hospitals. This enabled her to approach the subject with an open attitude, aiming to bracket other influences-excluding them through self-reflexivity. Associate Professor LJX who had published several qualitative research articles guided the study during the process. SHB, ZY, YAQ, and ZXS were involved in the study design and review of the analysis. All the researchers (including the main interviewer) had learned the qualitative research methods systematically and comprehensively.

### Aims

This study aims at exploring whether there are differences in psychological responses and coping styles among different gender nursing students after workplace violence and providing an in-depth analysis of the causes for the differences.

### Participants

A purpose sampling method (a non-probability sample selected based on the overall characteristics and study objectives) was used to select participants. From September 2020 to December 2021, nursing students were chosen from Guangzhou Medical University and Zunyi Medical University, in China.

Inclusion criteria: ① Undergraduate nursing students who were in internships ( ≧ 4 months)and the same academic year; ② Students must have experienced workplace violence and have described it clearly and in detail; ③ Students must have provided informed consent and participated voluntarily. Exclusion criteria: ① Students who were on personal leave, sick leave, or studying and training elsewhere; ② Students who were unable to self-report their gender.

Twenty-two participants were included, 11 males and 11 females, aged 20 to 23 (mean age: 21.5 years). No one declined participation in the study. This study determined whether a nursing student is a male or female nursing student based on their self-gender report. The internship time was from 4 to 11 months. The participants were numbered in turn according to the order of the interviews. Table 1 shows the participants’ general data.

**Table 1:** Demographic Information of Participants (X = 22)

Order	Gender	Age	Internship time (month)	Academic year
X1	Male	23	11	4th
X2	Male	22	11	4th
X3	Male	22	11	4th
X4	Male	22	11	4th
X5	Male	21	11	4th
X6	Male	22	11	4th
X7	Male	22	6	4th
X8	Male	23	5	4th
X9	Male	21	10	4th
X10	Male	21	10	4th
X11	Male	22	10	4th
X12	Female	21	11	4th
X13	Female	22	11	4th
X14	Female	22	11	4th
X15	Female	20	11	4th
X16	Female	22	11	4th
X17	Female	22	11	4th
X18	Female	21	4	4th
X19	Female	22	5	4th
X20	Female	20	5	4th
X21	Female	20	6	4th
X22	Female	21	7	4th

### Ethics

Local ethics institutions approved this study. Ethics information was publicly released and explained to the participants. All participants signed written informed consent.

### Data collection

Phenomenological methods of qualitative research were used to collect data in semi-structured in-depth interviews. Interviews were conducted via WeChat phone calls (including four researchers and one participant each time with one researcher responsible for interviewing, and the others for supplementing and recording). Appointments with the participant were scheduled in advance and the interviews were carried out in a quiet and private environment. Researchers explained the purpose and methods of the study before the interviews and pledged to protect participant privacy. The interviews were recorded by a whole process of synchronous recording, notes, and timely reminiscence during the interview, and participants’ views were clarified and confirmed to ensure accuracy. The interview times ranged from 30 to 40 min. The sample size was saturated when the information appeared repeatedly and there was no new content or topic. The study did not repeat the interviews.

### Data analysis

After the interview, a systematically written transcript of the interview was completed within 24 h by comparing the recording, notes, and reminiscence methods, and transcribing the interviewees’ narratives verbatim. The data were summarized, sorted, and analyzed according to the steps of Colaizzi of the qualitative content analysis [26]: ① Listen to the recording or take notes to find the feelings; ② Find out the meaningful statements; ③ Translate meaningful parts into general statements and encode the recurring views; ④ Derive implications from the meaningful and restatement sections, then gather together the encoded views; ⑤ Organize the exported meanings as themes, subject groups, and categories; ⑥ Connect themes to a complete narrative of research phenomena; ⑦ State the essential structure; ⑧ Return to the participants for verification and finally form the themes. Ultimately, 2 categories, 5 themes, and 14 subthemes were derived.

### Rigor

To ensure the rigor of this study, qualitative research was conducted in strict accordance with the standard when guaranteeing the participation of researchers in the whole process. All researchers were asked to systematically learn the qualitative research, avoiding subjective thinking. The research topic and results were determined under the guidance of an associate professor who has published many qualitative researches.

## Results

### Basic conditions of workplace violence experiences among nursing students

Twenty-two nursing interns (11 males and 11 females)were interviewed. Fifty-seven incidents of violence were reported. The main types of violence were verbal (50 cases), physical (3 cases), intentional ignoring (2 cases), psychological violence (1 case), and sexual harassment (1 case). Verbal violence, physical violence, intentional ignoring, and psychological violence were experienced by all the participants. One case of sexual harassment was witnessed by a female nursing student. All eleven males experienced verbal violence, and one of them was subjected to intentional ignoring, too. All eleven females experienced verbal violence, and three of them also were subjected to physical violence, one experienced intentional ignoring and one experienced psychological violence.

The abusers included mainly patients or their families (34 cases), clinical instructors (10 cases), doctors (11 cases), nursing workers (1 case), and a cleaner (1 case). The causes of experiencing violence mainly were associated with the operational ability and the theoretical knowledge of the nursing students were questioned and there was no timely communication. Besides, perpetrator factors were included as well. Table 2 shows the basic conditions of workplace violence among nursing students.

**Table 2:** Basic conditions of experiencing workplace violence of nursing students (X = 22)

Number	Gender	Frequency(times)		Source	Behaviors			Reasons		
X1	Male	3		Patient Doctor Cleaner	Verbal violence			Patient: Refused to cooperate. Doctor: Didn’t cooperate well with him. Cleaner: They were not willing to communicate with students.		
X2	Male	3		Doctors Instructor	Verbal violence			The operational ability and the theoretical knowledge were questioned.		
X3	Male	9		Doctors Instructor Patients or their families	Verbal violence			The operational ability and the theoretical knowledge were questioned.		
X4	Male	5		Doctors Instructors	Verbal violence			Doctors’ and instructors’ own factors, cooperation with students is low.		
X5	Male	3		Patient Doctor Instructor	Verbal violence Intentional ignoring			Perpetrators’ factors.		
X6	Male	2		Doctor Patient	Verbal violence			The operational ability was questioned by complicated medical procedures.		
X7	Male	1		Patient	Verbal violence			The ability was questioned and there was no timely communication.		
X8	Male	1		Patient	Verbal violence			The operational ability was questioned.		
X9	Male	1		Patient	Verbal violence			Refused to cooperate.		
X10	Male	3		Patients Doctor	Verbal violence			The operational ability was questioned by complicated medical procedures.		
X11	Male	1		Patient	Verbal violence			The ability was questioned and there was no timely communication.		
X12	Female	2		Patients	Verbal violence			The operating procedures were different.		
X13	Female	3		Patient Instructor Nursing worker	Verbal violence Intentional ignoring			The operational ability was questioned.		
X14	Female	5		Patients or their families Instructors	Verbal violence Psychological violence			The operational ability and the theoretical knowledge were questioned.		
X15	Female	1		Patient	Verbal violence			Complicated medical procedures lead to a difficult therapeutic process.		
X16	Female	3		Instructor Patients	Verbal violence Physical violence			Perpetrators’ factors.		
X17	Female	3		Patients	Verbal violence			The operational ability and the theoretical knowledge were questioned.		
X18	Female	1		Patient	Verbal violence			Their operational ability of them was questioned.		
X19	Female	2		Patients or their families	Verbal violence Physical violence			The operational ability and the theoretical knowledge were questioned.		
X20	Female	2		Patients or their families	Verbal violence Physical violence			Perpetrators’ factors.		
X21	Female	1		Patient	Verbal violence			Refused to cooperate.		
X22	Female	1		Patient	Verbal violence			There was no timely communication.		

**Table 3:** Themes of the psychological experience and coping styles of nursing students after workplace violence

**Psychological experience**			**Coping styles**	
Negative emotional experience	Low level of professional identity	Negative effect on self-efficacy	Responses to violence	Adjustment after violence
Anger		Impaired self-esteem	Explicate face-to-face	Self-adjustment
Fear		Self-doubt	Tolerance and avoidance	Talk to others
Grievance			Ask for help	Introspection and promotion
Sorrow				Understanding and acceptance
Helplessness				

From the interview data, 2 categories, 5 themes, and 14 subthemes were extracted. Three themes are regarding psychological experience: negative emotional experience, low level of professional identity, and negative effect on self-efficacy. Two themes are about coping styles: responses to violence and adjustment after violence. Table 3 summarizes the themes.

### Category 1: psychological experience–negative emotions, thoughts and feelings of nursing students experience violence

#### Theme 1: Negative emotional experience–all kinds of bad emotions of nursing students due to violence

##### Subtheme 1: Anger–situations in which different gender nursing students tend to feel angry

Nine male and six female nursing students expressed anger when they were faced with violence from doctors and patients. And the males were easier to feel angry when they were crudely ordered out to do something by doctors, because they thought that they were not being treated fairly, and they felt that their profession was not being respected. Male X6: “I once received a patient who needed to do a bedside electrocardiogram, and that should register first. However, a doctor directly asked me to ‘Do an electrocardiogram for him.’ I explained to him that he needed to register first, but the doctor roared, ‘Didn’t you hear what I said? Why go against me?’ I felt very angry and speechless at that moment. What power he was entitled to command me?” Female nursing students were more likely to be indignated when the work that they were able to complete was questioned by the patient in comparison to males. Because females thought that patients should not arbitrarily question anybody when they completely do not understand others’ professional ability, which was uncivilized behavior. Female X19: “One time I observed that a patient’s nose feed pump seemed to be motionless, I was ready to inspect it. The patient directly pushed me away and said, ‘You may be unskilled for this, ask your instructor to do it.’ I was very speechless, why didn’t allow me to explain? How did he know that I wouldn’t? I was just very angry so much.”

##### Subtheme 2: Fear–situations in which different gender nursing students tend to feel fear

Two male and one female student said they felt afraid after experiencing verbal violence from patients. Two males expressed that they feared the patients’ uncivilized behavior would aggravate and put them in more danger or be criticized by an instructor. Male X7: “I failed to puncture, the patient verbally attacked me, I was afraid that he would escalate, and the instructors’ condemnations.” However, the female said that the patient’s malice would make her afraid to work for fear of making mistakes. Female X14: “The patient found that my name tag shows that I am an intern, and refused me to do any operations, I was afraid that I would cause the patient’s dissatisfaction if I made a mistake.”

##### Subtheme 3: Grievance–situations in which different gender nursing students tend to feel wronged

Two males and eight females felt wrong when they experienced verbal or physical violence from doctors or patients. Just two males felt wronged when they were despised by the doctors because of their questioned operational ability. Male X11: “One day I was assigned to cooperate with a small operation, but I was not very familiar with the medical apparatuses and the cooperation with doctors was not so satisfactory. The doctor said, ‘Do you joke with me?’ The attitude was so scornful. I was so wrong that I didn’t understand why he had such an attitude.” Nevertheless, the females were more likely to feel wronged when experienced verbal and physical violence from patients. Female X22: “There was an autistic child. I was particularly careful and very kind to him. But when I injected him, he kicked and cursed me. I felt some grievance at that time.”

##### Subtheme 4: Sorrow–situations in which different gender nursing students tend to feel sad

Three males and three females said they would feel sad after experiencing verbal violence from doctors, instructors, and patients. Males were more likely to be sad when they were disgusted by their instructors for they were less familiar with the environment and operations. Male X3: “I just changed the department, so there were some operations that I was not very skilled in. However, the instructor did not train something to me patiently. I just felt a little lost and sad.” And the females were more likely to be sad when they were blamed by patients for unsuccessful punctures. Female X20: “I failed to puncture, the patient scolded me that I was not professional. He was so impatient, so I had to apologize, and felt lost and depressed.”

##### Subtheme 5: Helplessness– situations in which different gender nursing students tend to feel helpless

Three male and one female student expressed that they felt helpless when they knew that the instructors slandered them without any reason. They were confused by the occurrence of this bad situation. On this issue, male nursing students have the same view as females. Male X4: “I think if I did not good enough, the instructor should point out to me face to face and guide me to correct, rather than slandering me. I felt very helpless.” Female X16: “Some instructors didn’t respect students, they liked to gossip about the students, I didn’t like this behavior and felt very speechless.”

#### Theme 2: Low level of professional identity–nursing students experiencing workload violence feel that the nursing profession is not highly respected and has low status in society

Six males and five females stated that they would feel a low social status and disrespected after several exposures to workplace violence, and it led to a reduced level of professional identity assessment. This view is no tremendous difference between males and females. Male X5: “There is a big gap between nursing and clinical medicine, the patient’s attitudes toward doctors and nurses were different. Nursing was not only looked down upon by the outside world but also by doctors…” Female X13: “I felt so inferior, even though nursing is so unimportant. It has very little social status and is always in a passive position. If you were not satisfied, you could only endure it, otherwise, you would have complained, I felt bad and wanted to change careers later.”

#### Theme 3: Negative effect on self-efficacy–the workload violence leaves nursing students low self-esteem and skeptical about their professional competence

##### Subtheme 6: Impaired self-esteem–situations in which different gender nursing students tend to have low self-esteem

One male nursing student thought that his self-esteem was affected after being belittled by doctors, and he thought that he should not be disdained. Male X2: “I was just an intern, a newcomer in this department, and was not very familiar with many aspects, Why the doctor was so impatient with me? I felt very inferior and ashamed.” There were no female students who felt that their self-esteem was impaired in our interviews. It may be related to the difference in the forms of violence and the perpetrators.

##### Subtheme 7: Self-doubt–situations in which different gender nursing students tend to feel self-doubt

Two male nursing students expressed self-doubt and a lack of self-confidence after their operational ability was questioned several times, and the views were the same as one female student. Male X8: “After experiencing violence, I became less and less confident, sometimes even doubting my ability. I was hesitant about some operations because of tension and I was feared to make mistakes.” Female X15: “In fact, I could do many operations skillfully, but when I experienced verbal abuse from patients, I started getting doubts myself. Moreover, my work state was affected, I seemed to make more mistakes such as forgetting to check the patient’s name, etc.”

### Category 2: Coping styles–nursing student response during and after violence

#### Theme 4: Responses to violence–situations in which different gender nursing students tend to take the coping styles in case of violence

##### Subtheme 8: Explicate face-to-face–situations in which different gender nursing students chose face-to-face confrontation of violence

Seven male and three female nursing students explicated face to face with patients when they were able to complete some operations or they did not make mistakes. The males would bravely expound on the brutal patients. Male X1: “A drunk patient scolded us without any reason, and I responded directly ‘We would send you to the police station if you continued.” And the females would communicate with patients to explain and prove their operational ability when they were questioned by patients. Female X17: “When the patient refused me to do any operation on him. I did explain to him that I had been practicing for a long time and could do many aspects.”

##### Subtheme 9: Tolerance and avoidance–situations in which different gender nursing students tend to tolerate and avoid violence

Four males and six females chose to tolerate it to avoid the greater negative impact of the escalation of violence. One male student pointed out that he reported the situation to his superiors after experiencing verbal violence, but the feedback effect was not ideal, so he did not do that again. Male X9: “He was a doctor, but I was just an intern. Can I report the situation to my instructor? No one would care about these things, except myself.” Nevertheless, females chose to endure it for fear of being further hurt by the patient. Female X12: “When I went to give treatment to the patients and did nothing wrong, they said that I was very unprofessional. I chose to ignore him, just do not want to be pestered by him and avoid more serious problems.”

##### Subtheme 10: Ask for help–situations in which different gender nursing students tend to ask for assistance

Three male nursing students asked for help from their instructors when they were questioned by patients, but this method was more inclined to be chosen by six females to avoid the aggravation of the violence. Male X10: “The patient didn’t think I could do it well just because I was an intern, so if I didn’t get it right, he resented me with his words. I directly asked for help from my instructor to deal with it.” Female X18: “When I was resisted by patients, to prevent being complained, I directly found an instructor to deal with it.”

#### Theme 5: Adjustment after violence–situations in which different gender nursing students tend to take the coping styles after violence

##### Subtheme 11: Self-adjustment–situations in which different gender nursing students tend to self-adjust

More male nursing students released negative emotions by self-regulation after violence because they thought that their adjustment ability was excellent, although three females also chose this way. Male X4: “My adjustment ability was great, maybe it would affect my emotions at first, but it won’t last long. And I usually listened to music and went out for drinks with my friends.” The female students realized the goal of self-regulation by understanding the patients’ pessimism. FemaleX19: “I consoled myself that he was a cancer patient and was so sensitive to pain, therefore, it was normal for patients to lose emotional control.”

##### Subtheme 12: Talk to others–situations in which different gender nursing students tend to talk to others

Six male and five female students chose to share their violent experiences with classmates or friends. Because they expressed that they could share experiences. Male X11: “After that, I would talk to classmates and exchange experiences with each other, felt that the mood would be a little suddenly enlightened.” Female X21: “I shared my experiences with the members of the same group to avoid similar violence, and I could feel better.”

##### Subtheme 13: Introspection and promotion–situations in which different gender nursing students tend to introspect themselves and improve their capacity

Six males and three females did introspection after the violence, actively consolidated their theoretical knowledge, and strengthened the training of various operations. There was no great difference between males and females at this point. Male X5: “I introspected myself. I would review the theoretical knowledge when I was free and actively ask the instructors for some operational knowledge for strengthening my professional ability.” Female X13: “In fact, I did make the mistakes, the operation should be a little decisive, I should not hesitate. In addition, the operation of venipuncture was indeed not standardized enough, and the relevant knowledge needed to be consolidated in time.”

##### Subtheme 14: Understanding and acceptance–situations in which different gender nursing students tend to understand perpetrators’ behaviors

Two male nursing students could adjust themselves promptly and understand perpetrators’ behaviors with empathy because of good psychological endurance. And one female student stated she could understand patient violence due to the disease. Male X8: “In hindsight, the doctor was on the operating table for a day, so it was inevitable that he was very tired and the mood would be impatient, so the attitude was bad to me could be understandable.” Female X22: “Because this patient was an autistic child, I could understand his impatience. Well, although he kicked me, it was not serious, and his parents apologized to me, I thought it was nothing.”

In addition to the above findings, we found that some nursing students’ operational ability was questioned and they were unable to communicate and explain with the perpetrators, which increased the risk of experiencing violence. Although nursing students take courses on humanistic care and communication methods in school, these courses are not necessarily suitable for the clinical environment, after all, each department is different. On this issue, we suggest that hospitals or departments where nursing students conduct their practice can independently set up a course to help nursing students better adapt and respond to violence. This is also a new finding and worthy reference of the study. However, all of the above results of this study need to be further validated by researchers in other countries and regions. This also gave the researchers a revelation that the research methods in the implementation process also needed a process of continuous adjustment and improvement.

## Discussion

### Basic conditions analysis of violence among diverse gender nursing students

This study found that nursing students were subjected to frequent violence in the workplace. The main forms included verbal, physical, intentional ignoring, psychological, and sexual harassment. Males mainly experienced verbal violence and intentional ignoring, whereas females incurred the same experience but also physical violence, psychological violence, and sexual harassment. The reasons were related to the students’ operational ability and the theoretical knowledge was questioned, beside, it also included perpetrator factors. It was similar to the study which surveyed 129 nurse interns who experienced workplace violence [9]. Furthermore, it did not find differences in the sources and causes of violence. This observation was the same as the research which surveyed 150 nurse trainees [27]. However, we found that male nursing students more frequently experienced violence compared with female students. This finding differed from the study which conducted a cross-sectional survey of the frequency, sources, and forms of violence among 14 male and 93 female nurse interns [22]. Its results showed that patients or their families, instructors, and other clinical staff reported significantly more violence against female nursing students than against males. One explanation for the different results is that the two studies had different ratios of males and females. Another reason is that women are closely linked to nursing and have become a socially solidified stereotype in most Asian countries. Men who enter the nursing profession are vulnerable to patients and their families because their skills may be questioned [28]. Therefore, male nurses are more likely to experience workplace violence.

### Analysis of the psychological experience of different gender nursing students after workplace violence

Our findings found that nursing students were prone to negative emotions of anger, fear, grievance, sorrow, and helplessness after experiencing violence. These findings were similar to qualitative research conducted on the experiences of seven male and nine female nursing students [16]. From in-depth interviews, we found that male and female nursing students showed fear when they experienced verbal violence due to their operational ability being questioned and without timely communication. In addition, the students feared escalation of violence or a second violent act from their instructors. Male nursing students were more likely to show anger when faced with violence caused by perpetrator factors, whereas female students were more likely to feel grievance. These differences may be related to male traits. Aggressive traits of men make them prone to radical basic attitudes and emotions in the face of violence [21, 23, 29]. Women’s sentimental, weak, and humble qualities could lead to more negative basic psychological states in the face of violence [21, 23, 29]. In addition, Asian women tend to meekness, consideration, and obedience may also aggravate the grievance mood [30]. Males were more likely to develop low self-esteem and self-doubt after experiencing verbal violence from doctors and patients. Besides, one male nursing student had developed a month-long feeling of poor self-efficacy due to intentional ignoring by instructors. However, female nursing students were less frequent with this in the study. This may be related to male dominance and successful orientation traits. Men tend to show themselves in public to gain social approval. Thus, an experience of verbal violence and the implicit negative self-esteem effect will aggravate the impaired gender psychological status and lower self-efficacy of men [21, 23, 29]. And impaired self-esteem may remain for a long time if it is difficult to adjust quickly. Nursing students’ helplessness after experiencing instructors’ slander, and lower professional identity assessment after several exposures to violence had no great difference in the interviews.

### Analysis of coping styles after violence among different gender nursing students

This study showed that there were gender differences in student coping styles during and after violence. At the time of violence, students of different genders usually had different responses (endurance, avoidance, and seeking help) because of different gender characteristics and differences in abusers. We found that more male nursing students tended to communicate face to face when exposed to verbal violence from patients or their families, they usually tried to explain and communicate with the abusers, and they would use positive words to respond to the unreasonable patients. This response may be related to the masculine and tenacious qualities of the men, and the traditional Chinese cultural influences cause them to be more proactive and inclined to solve problems by themselves [21, 23, 25, 30]. Female nursing students were more inclined to seek help. This is the same as the research which reported that female nursing students usually asked for help to avoid more serious conflicts in the event of violence [31]. This behavior may be related to the subsidiary and effeminacy characteristics of women that cause them to be passive [21, 23–25, 30]. In addition, some nursing students chose to tolerate verbal violence from doctors and patients due to poor feedback after they had reported violence and they feared being further hurt.

We found that nursing students would choose ways such as talking with others and adopt self-regulation, introspection, self-improvement, understanding, and acceptance after experiencing violence. The male nursing students tended to self-regulate after the violence, such as listening to music and participating in sports. This strategy may be related to male self-control and a tough mental state, which cause them to be resilient to bad emotions [21, 23–25, 30]. However, half of the male nursing students in this study also talked with others. They explained that sharing experiences was conducive to the disappearance of negative emotions. This outcome may be related to the successful orientation characteristics of men and the “masculinity” endowed with inclusive, generous, and eclectic qualities in Orientalism [29]. Men actively open their minds to enrich their experience after violence. In addition, some males said that they could understand the verbal violence from doctors and patients when the violence was not excessive. Tenacity makes male nursing students rational when faced with violence [21,23,25,]. Thus, men can judge correctly the cause of the violence by analyzing the state and situation of the perpetrator, and they can explain and regulate to understand the behaviors of perpetrators. In addition, we found that the male nursing students were more active in introspection and improvement after violence compared with female nursing students. They strengthened professional knowledge and skills, which were mainly influenced by male dominance, conquering, and successful orientation traits. To obtain approval from doctors and patients, male nursing students actively analyzed the reasons for violence, found solutions, and improved their operation skills and theoretical knowledge [23].

### Recommendations

To reduce the adverse effects of violence, we recommend strengthening violent training to respond to violence. Nursing managers and educators need to avoid male students’ escalating violence when they are experiencing that and strengthen the psychological comfort and active guidance of female students. Besides, psychological assessments should be made on time to limit the negative effects of violence. Clinical instructors (nurses) should help nursing students (especially male nursing students) to better identify the aggressive patients and prevent violence. In addition, the instructors can teach more coping experiences to nursing students, help them (especially female nursing students) to better adjust their status, and pay more attention to their physical and mental health and learning status.

### Limitations

The sample size of this study was small and did not represent the psychological experience and coping styles of all nursing students who experience violent events. Only one interview for each participant could be insufficient for the richness of the data, chronological interviews or data triangulation can further exploration of the student’s feelings and reflections. In the process of data collation and analysis, the researchers might have transferred their subjective judgments and speculations. Besides, we did not include those who cannot actualize self-report gender.

## Conclusion

Nursing students were prone to bad emotional experiences from workplace violence, which affected their professional identity and self-efficacy. Male nursing students were more likely to become angry when exposed to violence, whereas female students were more likely to feel wronged. Male nursing students usually explicated face to face at the time of violence, whereas females tended to seek help. Male nursing students often took a self-regulation way to eliminate bad emotions and were more active in introspection and improvement after experiencing violence compared with female students. We suggest that managers need to take gender-specific, targeted measures to strengthen the psychological counseling and guidance of female nursing students and prevent male nursing students from taking radical steps to deal with perpetrators.

### Relevance to clinical practice

This study showed that nursing students mainly encounter vertical violence, which is a big obstacle to their study, life, and professional identity. In order to prevent bullying and violence during the practice of nursing students, training on prevention and response to violence should be provided according to the characteristics of nursing students of different genders. In addition, leaders should create a positive work atmosphere, boost team spirit of clinical staff, and show patience, love and empathy to nursing students. In addition, the leaders should build bridges between patients, families and students to help the patients better understand and support nursing students’ learning and work.

## Data Availability

The datasets produced during the current study are available from the corresponding author upon reasonable request.
